# Enhancer deletions of the *SHOX* gene as a frequent cause of short stature: the essential role of a 250 kb downstream regulatory domain

**DOI:** 10.1136/jmg.2009.067785

**Published:** 2009-07-02

**Authors:** J Chen, G Wildhardt, Z Zhong, R Röth, B Weiss, D Steinberger, J Decker, W F Blum, G Rappold

**Affiliations:** 1 Department of Molecular Human Genetics, University of Heidelberg, Heidelberg, Germany; 2 Bioscientia Institute of Medical Diagnostics, Ingelheim, Germany; 3 Lilly Research Laboratories, Eli Lilly and Company, Bad Homburg, Germany

## Abstract

**Background:**

Mutations and deletions of the homeobox transcription factor gene *SHOX* are known to cause short stature. The authors have analysed *SHOX* enhancer regions in a large cohort of short stature patients to study the importance of regulatory regions in developmentally relevant genes like *SHOX*.

**Methods:**

The authors tested for the presence of copy number variations in the pseudoautosomal region of the sex chromosomes in 735 individuals with idiopathic short stature and compared the results to 58 cases with Leri–Weill syndrome and 100 normal height controls, using fluorescence in situ hybridisation (FISH), single nucleotide polymorphism (SNP), microsatellites, and multiplex ligand dependent probe amplification (MLPA) analysis.

**Results:**

A total of 31/735 (4.2%) microdeletions were identified in the pseudoautosomal region in patients with idiopathic short stature; eight of these microdeletions (8/31; 26%) involved only enhancer sequences residing a considerable distance away from the gene. In 58 Leri–Weill syndrome patients, a total of 29 microdeletions were identified; almost half of these (13/29; 45%) involve enhancer sequences and leave the *SHOX* gene intact. These deletions were absent in 100 control persons.

**Conclusion:**

The authors conclude that enhancer deletions in the *SHOX* gene region are a relatively frequent cause of growth failure in patients with idiopathic short stature and Leri–Weill syndrome. The data highlights the growing recognition that regulatory sequences are of crucial importance in the genome when diagnosing and understanding the aetiology of disease.

Short stature is a developmental, multifactorial condition with a strong genetic component. Three per cent of the population are defined as being short. Short stature can have many different causes, a multitude of which are genetic. Several large scale genome wide association studies have recently determined variants that affect susceptibility to disease and identified a total of 54 validated single nucleotide polymorphism (SNP) variants with an influence on height.^1^ ^2^ ^3^ However, in total these large scale studies only pinpointed candidate genes of relatively minor effect within each locus, explaining only a small proportion of the phenotypic variance in the normal population, and thus accounting together for little more than 5% of our height (0.4 cm per “increasing” allele).

Although mutations in several genes have been reported that cause pronounced short or tall stature with a drastic effect on height (10–30 cm per “mutant” allele), this normally accounts for only a very small proportion of patients with short stature. So we are left with the fact that for the vast majority of short individuals with a height below −2 standard deviations scores (SDS), no causing defects are known, clinically termed as “idiopathic” short stature.^4^

One of the more prevalent causes of short stature was shown to derive from a defect in a developmental gene on the X chromosome, *SHOX* (short stature homeobox gene; MIM 312865). Defects in the coding region of *SHOX* have been demonstrated in individuals with different short stature syndromes including Leri–Weill, Langer and Turner syndrome (MIM 127300),^5^ ^6^ ^7^ ^8^ but mutations are also found in patients with idiopathic short stature (ISS) (MIM 300582).^9^

Regulatory elements residing either 5′ or 3′ of a gene can be sites of mutations in genetic disease and interfere with the normal expression of a gene. Some of these elements—defined as enhancers or repressors—can lie a considerable distance away from the coding portion of a gene.^10^ Enhancer elements can act upstream and downstream of a gene and function only in certain cell types. In concert with transcription factors that bind to these enhancer sequences, they play a role in achieving the appropriate level of gene expression. Defects in enhancer sequences have as yet only rarely been identified, and like promoter mutations, are considered to play only a relatively minor role in disease.

Comparative genomic analysis has previously identified evolutionarily conserved non-coding DNA elements, termed CNE, several hundred kilobases downstream of the *SHOX* gene in patients with Leri–Weill syndrome (LWS).^11^ ^12^ ^13^ ^14^ Three of these elements have subsequently been shown to act as enhancers in the chicken limb bud and five elements as enhancers in the neural tube.^15^ To establish the frequency and significance of long range regulatory elements also in individuals with idiopathic short stature (ISS), we have analysed the DNA of 735 patients with ISS and compared these results to 58 patients with a clinical manifestation of LWS and 100 controls.

## Subjects and methods

The study was conducted in accordance with the principles of the Declaration of Helsinki/Good Clinical Practice and was approved by the ethics committees of the participating institutions. Investigators obtained written informed consent from the participants’ parent(s) or legal guardian(s) before conducting study related procedures.

### Study subjects

We studied 740 unrelated pre-pubertal children with ISS from 14 countries: Belgium (n = 61), Canada (n = 39), Croatia (n = 34), Czech Republic (n = 87), France (n = 60), Germany (n = 144), Hungary (n = 29), India (n = 7), Netherlands (n = 2), Poland (n = 49), Russia (n = 6), Spain (n = 107), Turkey (n = 61), and USA (n = 54). Inclusion criteria were: chronological age ⩾3 years and pre-pubertal (males: genital stage Tanner 1 and testes ⩽2 ml; females: breast stage Tanner 1); height ⩽3rd centile of the local reference range or height ⩽10th centile with height velocity ⩽25th centile; bone age ⩽10 years for boys and ⩽8 years for girls; no growth hormone (GH) deficiency or GH resistance; no chronic disease; and no known growth influencing medications.

Our study also included 67 patients with LWS from eight different European countries: Germany (n = 37), Austria (n = 1), Croatia (n = 4), France (n = 10), Italy (n = 2), Netherlands (n = 8), Spain (n = 4), and Sweden (n = 1). The control cohort consisted of 100 individuals of European origin with normal stature. We used a combination of DNA sequencing, microsatellite analysis, fluorescence in situ hybridisation (FISH), and multiplex ligand dependent probe amplification (MLPA),^16^ to search for copy number variations in the proximity of *SHOX* in the pseudoautosomal region (PAR1). Details are given in the supplemental appendix.

## Results

The main focus of this study was to find out about the frequency of enhancer deletions in a large cohort of patients with ISS and compare the results to patients with LWS and control individuals. Patients that presented (point) mutations within the *SHOX* gene were excluded from the deletion analysis (five patients with ISS, nine patients with LWS), leaving altogether 735 patients with ISS, 58 patients with LWS, and 100 controls for the enhancer deletion analysis.

**Figure 1:**
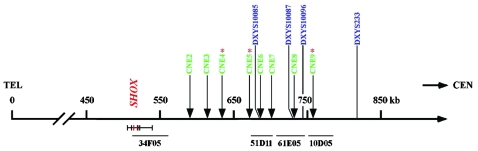
Schematic representation of the PAR1 region showing the position of evolutionarily conserved non-coding DNA elements (CNEs), microsatellites and cosmids that were used for deletion mapping.^15^ The *SHOX* gene is indicated in red and resides between 505.1 and 540.1 kb from the telomere (NCBI build 125, March 2006). The CNEs and microsatellites used for deletion mapping are shown above the scale bar. CNEs are indicated in green; CNE4, CNE5 and CNE9 with a star symbol have shown enhancer activity in the developing chicken limb bud.^15^ CNE3, CNE4, CNE5, CNE7 and CNE9 have previously shown enhancer activity in the chicken neural tube.^15^ Microsatellites are indicated in blue. The *SHOX* exons and the cosmids contig used for deletion mapping are shown below the scale bar. Cosmid 34F05 (LLN0YCO3′M34F05) includes the *SHOX* gene; Cosmid 51D11 (LLN0YCO3'M'51D11) includes CNE6, CNE7 and markers *DXYS10085*; Cosmid 61E05 (LLN0YCO3'M'61E05) includes CNE8 and markers *DXYS10087* and *DXYS10096*; Cosmid 10D05 (LLN0YCO3'M'10D05) includes CNE9. *TEL*, telomere; *CEN*, centromere.

To screen for enhancer deletions downstream of the *SHOX* gene, we carried out MLPA on 201 of the 735 ISS patients. These 201 patients were selected as they showed homozygosity with at least one of the microsatellite markers (*DXYS10085, DXYS10087*, and *DXYS10096*), in addition to the limb specific enhancer CNE9, all residing at a distance of 150–250 kb from the *SHOX* gene (fig 1).

We have also screened the 735 ISS patients for homozygosity of two markers in direct vicinity of the *SHOX* gene (DXYS201 and CAII),^14^ to pinpoint *SHOX* containing deletions. In all individuals where all two markers were homozygous, FISH analysis was carried out, and in 18 individuals a deletion of the *SHOX* gene was demonstrated by FISH. In these 18 individuals, MLPA was also carried out additionally as a further confirmation for the *SHOX* containing deletions. MLPA analysis was also carried out in DNA of 58 LWS patients who had been previously tested for the presence or absence of the *SHOX* gene and in DNA of 100 control individuals.

**Figure 2:**
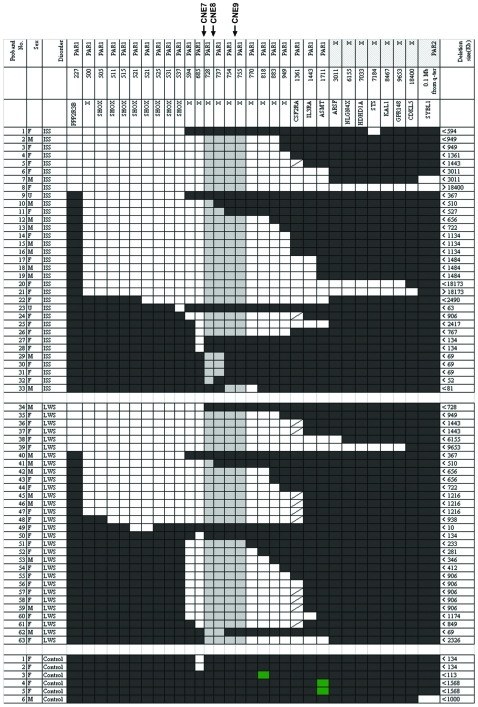
Mapping of 63 PAR deletions in patients with idiopathic short stature (ISS) and Leri–Weill syndrome (LWS) using the SALSA P018C and P018D-1 *SHOX* MLPA kit. Maximum deletion sizes are according to Ensemble Genome Browser coordinates (in kb) and are given on the right side. Blackened areas indicate the presence of two copies of the MLPA probe; white areas indicate the deletion of one allele; green areas indicate the presence of more than two copies of the MLPA probe; grey areas indicate the common minimal deletion interval in patients, and solidus areas indicate that the analyzed marker was non-informative. The ISS 24-33 and LWS 50-63 patients present cases with an intact *SHOX* region and downstream deletions. In the control population, two deletions of the locus at 685 Xptel, a duplication of locus at 818 Xptel, as well as two duplications of the *ASMT* gene at 1711 Xptel were found. Note also that in one patient (no.9) a deletion of the *STS* gene was identified.

In total, MLPA analysis demonstrated a deletion in the pseudoautosomal region of 33/201 tested ISS patients, with peaks at a ratio <0.5 as compared to normal controls (fig 2). Deletion sizes ranged between 52 kb and several megabases of DNA and no duplications were detected. Twenty-three of the 33 microdeletions included the *SHOX* gene. A comparison of the results to the control cohort showed that two of the deletions (no. 27 and 28; see fig 2) represent polymorphisms as they were also detected in two control individuals (fig 2). Among the 31 remaining microdeletions of the PAR region, eight ISS patients presented deletions including only the enhancer sequences, CNE7, CNE8 and/or CNE9 (no. 24–26, 29–33; fig 2). Three of the eight patients (no. 29–31) presented very small deletions of only the enhancer intervals CNE7 and CNE8, and in proband 32 only the CNE8 interval was deleted (CNEs as defined by Sabherwal *et al*^15^).

MLPA analysis also demonstrated a heterozygous deletion in the PAR region in 30/58 LWS patients, showing all the peaks at a ratio <0.5 as compared to normal controls. Among the 30 microdeletions, 16 deletions encompassed the *SHOX* gene, whereas 14 deletions were located downstream of the *SHOX* gene. The smallest deletion (no. 49, fig 2) included exons 4–5 of the *SHOX* gene (10 kb) and was only detectable by MLPA. A comparison with the controls demonstrated that the deletion in one of the patients (no. 50) represents a polymorphism. Among the 29 microdeletions of the PAR1 region in LWS patients, one patient (no. 62) included only the enhancer interval CNE7 and CNE8.

**Figure 3:**
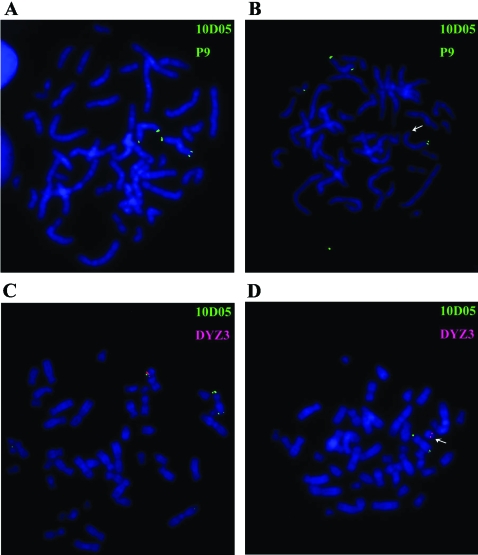
FISH analyses of patients with idiopathic short stature. The CNE9-containing cosmid 10D05 (LLN0YCO3'M'10D05) was used as a probe for FISH analysis. (A) Metaphase chromosomes from a female control with FISH signals on both X chromosomes. (B) Metaphase chromosomes from a female patient (no. 24) with a deletion on one of the chromosomes (arrows). (C) Metaphase from a male control with FISH signals on both X and Y chromosomes. (D) Metaphase from a male patient (no. 33) with a deletion on the Y chromosome (arrows) but not on the X chromosome. A reference probe residing on the long arm of the X-chromosome (cosmid P9) and a Y centromeric probe (*DYZ3*) were used as controls. The green signals indicate cosmids 10D05 and P9 and hybridised to opposite ends of the X chromosome; the red signal indicates probe *DYZ3*.

Altogether, we identified 63 persons with copy number variations in the PAR region by screening 793 patients with short stature (735 with ISS and 58 with LWS). Sixty of these deletions were absent in a series of 100 control persons, while three were also found in the control group and had to be judged as normal variants (no. 27, 28, 50). To confirm these results with an independent method, we carried out FISH on chromosomes of several patients with small deletions, where metaphase spreads were available (no. 24–26, 29–33, 62). In all cases, deletions were confirmed by FISH analysis using the cosmids 51D11, 61E05 and 10D05 with a map location between 670–780 kb from the telomere (fig 3).

To further correlate the significance of one or more of the enhancer CNE elements in *SHOX* downstream deletions, a detailed analysis of the deletion boundaries was carried out in seven ISS cases (no. 27–33) and one LWS case (no. 62) by SNP and microsatellite analysis (supplemental fig). The smallest deletion was confined to ⩽2 kb in proband 27 and 28 and represents a polymorphism; proband 33 had a deletion of ⩽80 kb. In three individuals with ISS and one individual with LWS, a deletion interval smaller than 60 kb was detected that included the CNE7 and CNE8 enhancer interval. The deletion in ISS proband 32 was the smallest detectable functional variant in this series and included only the CNE8 interval.

## Discussion

**Figure 4:**
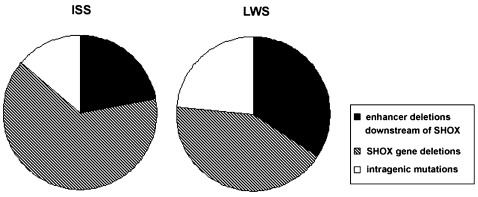
Summary of the spectrum of different *SHOX* mutations in patients with idiopathic short stature (ISS) and Leri–Weill syndrome (LWS). From all studied 735 unrelated patients with ISS, 31 are caused by *SHOX*-related deletions. Eight presented deletions proximal to *SHOX* (black) and 23 presented a *SHOX* gene deletion (hatched). A further five patients with ISS presented an intragenic mutation (white; 4 missense, 1 frameshift mutation; data not shown), which were initially excluded from the deletion study (total number of patients with ISS: 740). In 67 unrelated LWS, 38 are caused by *SHOX-* related defects: 13 presented a deletion downstream of *SHOX* (black), 16 presented a *SHOX* gene deletion (hatched) and nine presented an intragenic mutation (white; 5 missense, 3 nonsense, 1 frameshift mutation). The nine patients with intragenic mutations were initially excluded from the deletion study. In total, 4.9% of patients with ISS and 56.7% of patients with LWS are caused by point mutations or deletions of *SHOX* or its regulatory regions.

This study was performed to establish a comprehensive view of copy number variations in the pseudoautosomal region of the human sex chromosomes in 735 patients with idiopathic short stature. The pseudoautosomal region represents a block of sequence identity shared between the X and Y chromosomes, flanked by the telomere. It is characterised by an elevated CG content, abundant *Alu* repeats, and one of the highest recombination rates in the human genome.^17^ Deletions in the pseudoautosomal region were identified in 33 of the 735 screened ISS patients (4.5%); 31 of these were not found in the screened controls. Deletions in the PAR1 region were also identified in 30 of the 58 screened LWS cases and in three of the analysed controls. As we were interested in the level and significance of enhancer deletions in patients with idiopathic short stature with regard to the overall frequency of detected mutations/deletions, the incidence of enhancer deletions of 22% was an unexpected finding, while this proportion was even higher at 34% in patients with LWS. Figure 4 gives a summary of all the *SHOX* gene deletions, the intragenic mutations and downstream enhancer deletions in the analysed patients with ISS and LWS. It is very likely that these high incidences are still an underestimate, as we cannot rule out the possibility that some cases have been overlooked due to point mutations in enhancer sequences or deletions in other parts of this region.

Do cis-regulatory mutations in ISS or LWS make a qualitatively different contribution to disease? LWS is clinically defined by a shortened middle portion of the limb (mesomelia) leading to short stature and an abnormal alignment of the radius and ulna at the wrist. The Madelung deformity typically develops at puberty and females are generally more severely affected than males. Other clinical features include a high muscle mass and high body mass index.^14^ ^18^ ^19^ As cis-regulatory deletions in our analysis occur more frequently in patients with LWS compared to ISS, it is possible that enhancer deletions may lead to more pronounced phenotypes, as exemplified in the more striking phenotype of LWS.

The deletion of the limb specific enhancer CNE9 has been previously shown to be an important cis-controlling element in SHOX haploinsufficiency.^11^ ^12^ ^13^ ^15^ This study confirms that CNE9 probably represents the most important enhancer in the proximal SHOX region, but also has revealed that CNE8 together with CNE7, and also the deletion of the CNE8 interval alone, can be identified in patients with short stature as well as skeletal defects (no. 29–32, 62; supplemental fig). Alternatively, it also cannot be totally ruled out that the deletion of the CNE7 and CNE8 intervals may have only a minor effect, as this interval was absent in the sequence data of one recently sequenced individual; however, pedigree data were not available and SNP and microsatellite analysis, MLPA and FISH was not carried out.^20^ It is also possible that this part of the sequence is still inaccurate or incomplete. We also know that the phenotype in patients can be very variable (intra- and interfamiliar) and that there are individuals with SHOX deficiency and normal stature.^12^ ^14^

Long range transcriptional control by enhancer sequences have been identified mostly through the analysis of patients with genetic malformations and found to reside up to hundreds of kilobases away from the genes they control.^10^ Three examples of long range control in limb development include enhancers in the vicinity of the mammalian *Hoxd* cluster^21^ and the *Sox9* gene, defects in which cause campomelic dysplasia, a dominant skeletal malformation syndrome often associated with XY sex reversal, or Pierre Robin sequence, an important subgroup of cleft palate.^22^ ^23^ ^24^ Chromosome rearrangements which separate the genes from their conserved non-coding DNA elements have only been reported in a small minority of cases. The *PAX6* and *SHH* position effect cases are further good examples of the separation of cis-regulatory elements.^10^ Given the findings on enhancer significance on key regulatory master genes in the literature, the high level of enhancer deletions in the *SHOX* gene is to our knowledge unprecedented in the human genome.

The conserved non-coding elements in the vicinity of the *SHOX* gene are embedded in a region with characteristics of a gene desert. Deletions encompassing more proximal or more distal pseudoautosomal genes have been detected in a number of patients in our study, but this does not lead to obvious phenotypic consequences. The same holds true for a duplication of the *ASMT* gene which was found in two normal controls. An important diagnostic issue has, however, been raised, related to the observation that five female probands carry deletions extending into the X-specific region of the X chromosome. While these females only present short stature, their male babies could also suffer from mental retardation, chondrodysplasia punctata, ichthyosis, and Kallmann syndrome,^25^ depending on their exact deletion size and which X chromosomal genes are missing. For these individuals, prenatal diagnosis might represent an option.

In summary, we have applied DNA sequencing, microsatellite, FISH and MLPA analysis to detect DNA rearrangements in a large cohort of patients with idiopathic short stature of different ethnic origin. We have provided strong evidence that cis-regulatory deletions can contribute to the patient’s condition to an unexpectedly high degree. These data also re-emphasise that regulatory input can be crucial for developmental genes, most notably genes with a dynamic expression pattern. Lastly, our findings also help to optimise diagnostic options for individuals with SHOX haploinsufficiency and thus may have immediate value for patients with idiopathic short stature.

## References

[1] Gudbjartsson DF Walters GB Thorleifsson G Stefansson H Halldorsson BV Zusmanovich P Sulem P Thorlacius S Gylfason A Steinberg S Helgadottir A Ingason A Steinthorsdottir V Olafsdottir EJ Olafsdottir GH Jonsson T Borch-Johnsen K Hansen T Andersen G Jorgensen T Pedersen O Aben KK Witjes JA Swinkels DW den Heijer M Franke B Verbeek AL Becker DM Yanek LR Becker LC Tryggvadottir L Rafnar T Gulcher J Kiemeney LA Kong A Thorsteinsdottir U Stefansson K Many sequence variants affecting diversity of adult human height. Nat Genet 2008;40:609–15 18391951 10.1038/ng.122

[2] Lettre G Jackson AU Gieger C Schumacher FR Berndt SI Sanna S Eyheramendy S Voight BF Butler JL Guiducci C Illig T Hackett R Heid IM Jacobs KB Lyssenko V Uda M, Diabetes Genetics Initiative, FUSION, KORA, Prostate, Lung Colorectal and Ovarian Cancer Screening Trial, Nurses’ Health Study, SardiNIA, Boehnke M Chanock SJ Groop LC Hu FB Isomaa B Kraft P Peltonen L Salomaa V Schlessinger D Hunter DJ Hayes RB Abecasis GR Wichmann HE Mohlke KL Hirschhorn JN Identification of ten loci associated with height highlights new biological pathways in human growth. Nat Genet 2008;40:584–91 18391950 10.1038/ng.125 PMC2687076

[3] Weedon MN Lango H Lindgren CM Wallace C Evans DM Mangino M Freathy RM Perry JR Stevens S Hall AS Samani NJ Shields B Prokopenko I Farrall M Dominiczak A, Diabetes Genetics Initiative, Wellcome Trust Case Control Consortium, Johnson T Bergmann S Beckmann JS Vollenweider P Waterworth DM Mooser V Palmer CN Morris AD Ouwehand WH, Cambridge GEM Consortium, Zhao JH Li S Loos RJ Barroso I Deloukas P Sandhu MS Wheeler E Soranzo N Inouye M Wareham NJ Caulfield M Munroe PB Hattersley AT McCarthy MI Frayling TM Genome-wide association analysis identifies 20 loci that influence adult height. Nat Genet 2008;40:575–83 18391952 10.1038/ng.121 PMC2681221

[4] Cohen P Rogol AD Deal CL Saenger P Reiter EO Ross JL Chernausek SD Savage MO Wit JM, 2007 ISS Consensus Workshop participants Consensus statement on the diagnosis and treatment of children with idiopathic short stature. J Clin Endocrinol Metab 2008;93:4210–7 18782877 10.1210/jc.2008-0509

[5] Ellison JW Wardak Z Young MF Gehron Robey P Laig-Webster M Chiong W PHOG, a candidate gene for involvement in the short stature of Turner syndrome. Hum Mol Genet 1997;6:1341–7 9259282 10.1093/hmg/6.8.1341

[6] Belin V Cusin V Viot G Girlich D Toutain A Moncla A Vekemans M Le Merrer M Munnich A Cormier-Daire V *SHOX* mutations in dyschondrosteosis (Leri-Weill syndrome). Nat Genet 1998;19:67–9 9590292 10.1038/ng0198-67

[7] Shears DJ Vassal HJ Goodman FR Palmer RW Reardon W Superti-Furga A Scambler PJ Winter RM Mutation and deletion of the pseudoautosomal gene SHOX cause Leri-Weill dyschondrosteosis. Nat Genet 1998;19:70–3 9590293 10.1038/ng0198-70

[8] Kosho T Muroya K Nagai T Fujimoto M Yokoya S Sakamoto H Hirano T Terasaki H Ohashi H Nishimura G Sato S Matsuo N Ogata T Skeletal features and growth patterns in 14 patients with haploinsufficiency of SHOX: implications for the development of Turner syndrome. J Clin Endocrinol Metab 1999;84:4613–21 10599728 10.1210/jcem.84.12.6289

[9] Rao E Weiss B Fukami M Rump A Niesler B Mertz A Muroya K Binder G Kirsch S Winkelmann M Nordsiek G Heinrich U Breuning MH Ranke MB Rosenthal A Ogata T Rappold GA Pseudoautosomal deletions encompassing a novel homeobox gene cause growth failure in idiopathic short stature and Turner syndrome. Nat Genet 1997;16:54–63 9140395 10.1038/ng0597-54

[10] Kleinjan DA van Heyningen V Long-range control of gene expression: emerging mechanisms and disruption in disease. Am J Hum Genet 2005;76:8–32 15549674 10.1086/426833 PMC1196435

[11] Benito-Sanz S Thomas NS Huber C Gorbenko del Blanco D Aza-Carmona M Crolla JA Maloney V Rappold G Argente J Campos-Barros A Cormier-Daire V Heath KE A novel class of Pseudoautosomal region 1 deletions downstream of SHOX is associated with Leri-Weill dyschondrosteosis. Am J Hum Genet 2005;77:533–44 16175500 10.1086/449313 PMC1275603

[12] Huber C Rosilio M Munnich A Cormier-Daire V, French SHOX GeNeSIS Module High incidence of SHOX anomalies in individuals with short stature. J Med Genet 2006;43:735–9 16597678 10.1136/jmg.2006.040998 PMC2564573

[13] Fukami M Kato F Tajima T Yokoya S Ogata T Transactivation function of an approximately 800-bp evolutionarily conserved sequence at the SHOX 3′ region: implication for the downstream enhancer. Am J Hum Genet 2006;78:167–70 16385461 10.1086/499254 PMC1380216

[14] Rappold G Blum WF Shavrikova EP Crowe BJ Roeth R Quigley CA Ross JL Niesler B Genotypes and phenotypes in children with short stature: clinical indicators of SHOX haploinsufficiency. J Med Genet 2007;44:306–13 17182655 10.1136/jmg.2006.046581 PMC2597980

[15] Sabherwal N Bangs F Röth R Weiss B Jantz K Tiecke E Hinkel GK Spaich C Hauffa BP van der Kamp H Kapeller J Tickle C Rappold G Long-range conserved non-coding SHOX sequences regulate expression in developing chicken limb and are associated with short stature phenotypes in human patients. Hum Mol Genet 2007;16:210–22 17200153 10.1093/hmg/ddl470

[16] Schouten JP McElgunn CJ Waaijer R Zwijnenburg D Diepvens F Pals G Relative quantification of 40 nucleic acid sequences by multiplex ligation-dependent probe amplification. Nucleic Acids Res 2002;30:e57 12060695 10.1093/nar/gnf056 PMC117299

[17] Blaschke RJ Rappold G The pseudoautosomal regions, SHOX and disease. Curr Opin Genet Dev 2006;16:233–9 16650979 10.1016/j.gde.2006.04.004

[18] Ross JL Scott C Jr Marttila P Kowal K Nass A Papenhausen P Abboudi J Osterman L Kushner H Carter P Ezaki M Elder F Wei F Chen H Zinn AR Phenotypes Associated with SHOX Deficiency. J Clin Endocrinol Metab 2001;86:5674–80 11739418 10.1210/jcem.86.12.8125

[19] Flanagan SF Munns CF Hayes M Williams B Berry M Vickers D Rao E Rappold GA Batch JA Hyland VJ Glass IA Prevalence of mutations in the short stature homeobox containing gene (SHOX) in Madelung deformity of childhood. J Med Genet 2002;39:758–63 12362035 10.1136/jmg.39.10.758 PMC1734979

[20] Levy S Sutton G Ng PC Feuk L Halpern AL Walenz BP Axelrod N Huang J Kirkness EF Denisov G Lin Y MacDonald JR Pang AW Shago M Stockwell TB Tsiamouri A Bafna V Bansal V Kravitz SA Busam DA Beeson KY McIntosh TC Remington KA Abril JF Gill J Borman J Rogers YH Frazier ME Scherer SW Strausberg RL Venter JC The diploid genome sequence of an individual human. PLoS Biol 2007;5:e254 17803354 10.1371/journal.pbio.0050254 PMC1964779

[21] Spitz F Gonzalez F Duboule D A global control region defines a chromosomal regulatory landscape containing the HoxD cluster. Cell 2003;113:405–17 12732147 10.1016/s0092-8674(03)00310-6

[22] Bagheri-Fam S Barrionuevo F Dohrmann U Günther T Schüle R Kemler R Mallo M Kanzler B Scherer G Long-range upstream and downstream enhancers control distinct subsets of the complex spatiotemporal Sox9 expression pattern. Dev Biol 2006;291:382–97 16458883 10.1016/j.ydbio.2005.11.013

[23] Velagelati GV Bien-Willner GA Northup JK Lockhart LH Hawkins JC Jalal SM Withers M Lupski JR Stankiewicz P Position effects due to chromosome breakpoints that map approximately 900 kb upstream and approximately 1.3 Mb downstream of SOX9 in two patients with campomelic dysplasia. Am J Hum Genet 2005;76:652–62 15726498 10.1086/429252 PMC1199302

[24] Benko S Fantes JA Amiel J Kleinjan DJ Thomas S Ramsay J Jamshidi N Essafi A Heaney S Gordon CT McBride D Golzio C Fisher M Perry P Abadie V Ayuso C Holder-Espinasse M Kilpatrick N Lees MM Picard A Temple IK Thomas P Vazquez MP Vekemans M Crollius HR Hastie ND Munnich A Etchevers HC Pelet A Farlie PG Fitzpatrick DR Lyonnet S Highly conserved non-coding elements on either side of SOX9 associated with Pierre Robin sequence. Nat Genet 2009;41:359–64 19234473 10.1038/ng.329

[25] Ballabio A Bardoni B Carrozzo R Andria G Bick D Campbell L Hamel B Ferguson-Smith MA Gimelli G Fraccaro M Maraschio P Zuffardi O Guioli S Camerino G Contiguous gene syndromes due to deletions in the distal short arm of the human X chromosome. Proc Natl Acad Sci U S A 1989;86:10001–5 2602357 10.1073/pnas.86.24.10001 PMC298630

